# The Impact of Alcohol Use on Drop-out and Psychological Treatment Outcomes in Improving Access to Psychological Therapies Services: an Audit

**DOI:** 10.1017/S1352465817000819

**Published:** 2018-02-26

**Authors:** J.E.J. Buckman, I. Naismith, R. Saunders, T. Morrison, S. Linke, J. Leibowitz, S. Pilling

**Affiliations:** 1 Research Department of Clinical, Educational and Health Psychology, University College London, Gower Street, London WC1E 6BT; 1a iCope – Camden and Islington Psychological Therapies Services, Camden and Islington NHS Foundation Trust, St Pancras Hospital, 4 St Pancras Way, London NW1 0PE; 2 Universidad de los Andes, Cra. 1 #18a-12, Bogotá, Colombia; 3 Research Department of Clinical, Educational and Health Psychology, University College London, Gower Street, London WC1E 6BT; 4 Hertfordshire Wellbeing Service, Hertfordshire Partnership University Foundation Trust, The Colonnades, Beaconsfield Road, Hatfield, Hertfordshire AL10 8YE; 5 Complex Depression, Anxiety and Trauma Service (CDAT), Camden and Islington NHS Foundation Trust, St Pancras Hospital, 4 St Pancras Way, London NW1 0PE; 6 iCope – Camden and Islington Psychological Therapies Services, Camden and Islington NHS Foundation Trust, St Pancras Hospital, 4 St Pancras Way, London NW1 0PE; 7 Research Department of Clinical, Educational and Health Psychology, University College London, Gower Street, London WC1E 6BT; 7a iCope – Camden and Islington Psychological Therapies Services, Camden and Islington NHS Foundation Trust, St Pancras Hospital, 4 St Pancras Way, London NW1 0PE

**Keywords:** alcohol misuse, cognitive therapy, anxiety disorders, depression, psychological therapies

## Abstract

**Background**: The impact of alcohol use disorders (AUD) on psychological treatments for depression or anxiety in primary care psychological treatment services is unknown. **Aims**: To establish levels of alcohol misuse in an Improving Access to Psychological Therapies (IAPT) service, examine the impact of higher risk drinking on IAPT treatment outcomes and drop-out, and to inform good practice in working with alcohol misuse in IAPT services. **Method:** 3643 patients completed a brief questionnaire on alcohol use pre-treatment in addition to measures of depression, anxiety and functioning. Symptom and functioning measures were re-administered at all treatment sessions. **Results**: Severity of alcohol misuse was not associated with treatment outcomes, although those scoring eight or more on the AUDIT-C were more likely to drop out from treatment. **Conclusions:** IAPT services may be well placed to offer psychological therapies to patients with common mental disorders and comorbid AUD. Patients with AUD can have equivalent treatment outcomes to those without AUD, but some higher risk drinkers may find accessing IAPT treatment more difficult as they are more likely to drop out. Alcohol misuse on its own should not be used as an exclusion criterion from IAPT services. Recommendations are given as to how clinicians can: adjust their assessments to consider the appropriateness of IAPT treatment for patients that misuse alcohol, consider the potential impact of alcohol misuse on treatment, and improve engagement in treatment for higher risk drinkers.

## Introduction

Alcohol use disorders (AUD) are highly comorbid with psychiatric conditions, with up to 85% of patients seen in alcohol disorder services reporting a comorbid psychiatric condition (Weaver et al., 2003). This comorbidity contributes to patients being passed between specialist mental health services and specialist alcohol services, as well as a higher rate of patients declining treatment or failing to access treatment. For those who do access treatment, these issues can give rise to significant treatment delays and to poorer outcomes (e.g. Fonseca et al., 2012).

### Alcohol use and Improving Access to Psychological Therapies services

In the UK, IAPT services receive approximately 1.4 million referrals nationwide for patients with depression or anxiety each year (NHS Digital, 2016). IAPT services do not routinely offer treatment to address AUD, but national guidance for IAPT clinicians suggests that patients with comorbid substance misuse should have access to evidence-based psychological treatments for depression and anxiety (IAPT, 2012). There have been few studies of the impact of alcohol misuse on outcomes for patients in IAPT services, although in one of the few studies ex-members of the UK Armed Forces attending IAPT services had significantly more symptoms of depression post-treatment if they were misusing drugs or alcohol compared with those not misusing substances (Giebel et al., 2014). However, the study has limited applicability to inform guidelines for alcohol use across IAPT services as only 0.015% of all patients referred to IAPT services are identified as ex-Armed Forces personnel (NHS Digital, 2016) and the prevalence of excessive alcohol consumption in the ex-Armed Forces population is considerably higher than in the general population (Fear et al., 2007). Furthermore, the authors were not able to control for many relevant confounding factors unique to this population, particularly the circumstances of their discharge from the Armed Forces (e.g. Buckman et al., 2013).

Given that high drop-out rates are seen in specific substance abuse treatment programs (Ryan et al., 1995) and higher rates of alcohol misuse have been associated with drop-out from psychological therapies such as imaginal exposure with post-traumatic stress disorder (PTSD) patients (Van Minnen et al., 2002) it is plausible that the impact of alcohol misuse on treatment outcomes in the IAPT population might be affected by the ability of patients with AUD to engage in and complete therapy.

### Aim

Data on alcohol consumption are not routinely collected in IAPT services, so the prevalence of alcohol misuse among IAPT patients is largely unknown. This article describes an audit into levels of alcohol use in a London IAPT service over a one-year period to determine levels of AUD in the service and explore the impact of AUD on drop-out from therapy and on treatment outcomes. The results of the audit are used to develop advice for IAPT clinicians working with patients with AUD.

## Method

### Participants

All patients referred to the IAPT service between 1 July 2013 and 30 June 2014 that had an assessment were asked to complete a brief measure of alcohol use, and those that completed it were included in the audit.

### Measures

The *Alcohol Use Disorders Identification Test-Consumption* (AUDIT-C) (Bush et al., 1998) is used to identify high-risk drinking behaviour and to screen for alcohol misuse disorders. Two different cut-offs were used: (i) scoring ≥6, which has a sensitivity of 88% and specificity of 84% for men, and a sensitivity of 68% and specificity of 91% for women in identifying higher risk drinkers in the UK primary care population (Aertgeerts et al., 2001), and (ii) scoring ≥8, which is considered the optimal cut-off for identifying higher risk drinking among those seeking advice in an online study about how to cut down their drinking (Khadjesari et al., 2017). These two cut-offs were used as they have considerably higher sensitivity and specificity in identifying higher risk drinkers than the typical cut-offs of ≥4 in men and ≥3 in women (Aertgeerts et al., 2001), and as IAPT services receive a mix of online self-referrals and referrals from primary care physicians/general practitioners they represent appropriate ways of identifying higher risk drinkers in these settings. Identifying higher risk drinkers at assessment might affect whether or not IAPT services consider their treatments appropriate for the patient, thus impacting upon whether or not the patient receives IAPT treatment or is referred on to alcohol disorder services.

The *Patient Health Questionnaire 9-item Version* (PHQ-9) (Kroenke et al., 2001) is a measure of depressive symptoms. Caseness is defined as a score of ≥10.

The *Generalized Anxiety Disorder 7-item Scale* (GAD-7) (Spitzer et al., 2006) is a measure of general anxiety symptoms. Caseness is defined as a score of ≥8.

The *IAPT Phobias Scale* (IAPT, 2011) is used to identify types of anxiety not covered by GAD-7, particularly social phobia, agoraphobia and specific phobias.

The *Work and Social Adjustment Scale* (WSAS) (Mundt et al., 2002) is a five-item measure of the impact of mental health problems on functioning: at work; at home; during social and private leisure activities; and in family relationships.

### Procedure

All patients assessed in the IAPT services completed the PHQ-9, GAD-7, Phobia Scale and WSAS as part of their routine assessment. They were also asked to complete the AUDIT-C, and those that did were included in the audit. Inclusion in the audit did not affect treatment within the IAPT service, so those that received treatment completed the symptom and functioning measures at every session whether or not they were included in the audit.

### Analysis

#### Primary outcomes

(1) Drop-out: participants were classified as dropped out or not based on the reason for discharge from the services recorded by the treating clinician. Participants who were discharged after their initial assessments without receiving any treatment, including those referred to other services without receiving IAPT treatment, were excluded from these analyses.
(2) Recovery: patients are considered recovered if they scored in caseness on either the PHQ-9 or GAD-7 pre-treatment, and score below caseness on both measures post-treatment (NHS England, 2014). This definition requires that patients have a minimum of two treatment sessions.

#### Secondary outcomes

Reliable and clinically significant change (RCSC): on the PHQ-9 this was defined as a change from caseness to non-caseness and a change of ≥5 points pre–post treatment (McMillan et al., 2010). On the GAD-7 this was defined as a change from caseness to non-caseness and a change of ≥4 points pre–post treatment (Gyani et al., 2013). Participants were then categorized according to whether or not they had reached RCSC on either measure.

#### Explanatory factors

Demographics, treatment-related factors (such as the number of sessions attended and whether or not patients were prescribed pscyhotropic medications), and pre-treatment scores on the symptom measures, functioning measures and the AUDIT-C were treated as explanatory factors to consider their relationship with primary and secondary outcomes. An additional variable described whether or not patients were engaged in treatment focused on symptomatic improvement. Although most patients seen in the service engage in treatment typically considered to be recovery focused, some patients are engaged in other types of treatment such as stabilization for patients with PTSD. These patients are excluded from analyses of the outcome related to recovery as it is not an expected outcome of the treatment; however, they can still achieve symptomatic improvements so are included in analyses related to RCSC.

### Statistical analyses

All analyses were conducted using STATA version 13.0 (StataCorp, 2013). Logistic regression models were built to consider whether either of the two cut-offs on the AUDIT-C at baseline were related to the primary and secondary outcomes after controlling for likely confounding factors based on *a priori* assumptions (such as symptoms of depression and anxiety would confound the relationship between AUDIT-C scores and outcome) and the strength of univariable associations. Variables were kept in regression models based on these factors or whether or not they improved the overall model based on likelihood ratio tests.

## Results

### Descriptive statistics

A total of 5330 patients were assessed in the IAPT services over the audit period. Of these, 3643 (68.9%) completed the AUDIT-C at assessment and so were included in this audit. Baseline characteristics of both the audit sample and patients who did not complete the AUDIT-C are presented in Table 1. Reasons for not completing the AUDIT-C were not recorded; however, 115 (6.8%) of those not included were patients of another IAPT service that co-facilitates some group therapies with the audit service, and 38 (2.25%) were seen in partner organizations. Patients of these other services were not asked to complete the AUDIT-C so could not be included in the present audit but are kept in the comparison sample as their data are counted in the national reporting of the primary and secondary outcomes for the service as a whole. For a higher proportion of those not completing the AUDIT-C compared with those that did complete it (6% compared with 3.5%), the IAPT service was not deemed the most suitable location for treatment so they were referred on before entering treatment. Additionally, a higher proportion (19.26% compared with 15.95%) were referred on to other services for treatment after having had at least one treatment session. Those that took part in the audit were slightly younger, were less likely to have ethnicity recorded as ‘other’, were more likely to be employed, were less likely to have a diagnosis categorized as ‘other’, were less likely to have been prescribed psychotropic medications, and had marginally lower pre-treatment scores on the PHQ-9, WSAS and all three IAPT Phobia Scale questions.

**Table 1.** Comparison of those included and not included in the audit

		Not in audit *n* (%)	In audit *n* (%)	OR (95% CI), *p*-value or *t*-test, *p*-value
		1687 (31.65)	3643 (68.35)	
**Pre-treatment factors**				
Age	Mean (*SD*)	39.58 (13.59)	38.16 (12.85)	*t* (4775) = 3.33, *p* = .0009
	Range	17 – 87	17 – 89	
Gender	Female	935 (66.12)	2189 (65.99)	0.99 (0.87 – 1.13), *p* = .93
	Male	479 (33.88)	1128 (34.01)
Ethnicity	White	761 (71.59)	2048 (75.24)	1.0
	Black	96 (9.03)	224 (8.23)	0.87 (0.67 – 1.12), *p* = .27
	Mixed	88 (8.28)	194 (7.13)	0.82 (0.63 – 1.07), *p* = .14
	Asian	53 (4.99)	154 (5.66)	1.08 (0.78 – 1.49), *p* = .64
	Other	65 (6.11)	102 (3.75)	0.58 (0.42 – 0.81) *p* = .0009
Employment Status	Employed	769 (51.30)	2217 (62.08)	1.0
	Homemaker	46 (3.07)	124 (3.47)	0.93 (0.66 – 1.34), *p* = .71
	Retired	82 (5.47)	134 (3.75)	0.57 (0.43 – 0.76), *p* = .0001
	Unemployed	305 (20.35)	626 (17.53)	0.71 (0.60 – 0.84), *p* < .0001
	Long term sick or disabled	297 (19.81)	470 (13.16)	0.55 (0.46 – 0.65), *p* < .0001
Receiving statutory sick pay	Yes	62 (4.20)	151 (4.27)	1.02 (0.75 – 1.38), *p* = .91
	No	1414(95.80)	3386 (95.73)	1.0
Primary diagnosis	Depression	409 (45.14)	1129 (45.93)	1.0
	Anxiety	286 (31.57)	833 (33.89)	1.28 (0.96 – 1.72), *p* = .098
	Mixed anxiety and depression	67 (7.40)	237 (9.64)	1.06 (0.89 – 1.26), *p* = .55
	Severe mental illness	14 (1.55)	35 (1.42)	0.91 (0.48 – 1.70), *p* = .76
	Other	130 (14.35)	224 (9.11)	0.62 (0.59 – 0.80), *p* < .001
Prescribed psychotropic medications	Yes	623 (52.13)	1418 (43.36)	1.0
	No	572 (47.87)	1852 (56.64)	1.42 (1.24 – 1.63), *p* < .0001
IAPT treatment	Yes	1628 (96.50)	3508 (96.29)	0.94 (0.69 – 1.29), *p* = .71
	No	59 (3.50)	135 (3.71)	1.0
Number of attended treatment sessions	Mean (*SD*)	4.95 (4.76)	4.89 (4.95)	*t* (4126) = –.38, *p* = .70
PHQ-9 score	Mean (*SD*)	14.90 (6.89)	14.37 (6.61)	*t* (5156) = 2.61, *p* = .0091
PHQ-9 caseness	Yes	1164 (75.49)	2688 (74.34)	0.94 (0.82 – 1.08), *p* = .38
	No	378 (24.51)	928 (25.66)	1.0
GAD-7 score	Mean (*SD*)	13.03 (6.05)	12.99 (6.27)	*t* (5133) = 0.19, *p* = .85
GAD-7 caseness	Yes	1334 (81.74)	2774 (79.28)	0.85 (0.74 – 0.99), *p* = .040
	No	298 (18.26)	725 (20.72)	1.0
WSAS score		20.16 (10.46)	18.42 (9.82)	*t* (4761) = 5.43, *p* < .0001
Phobia Scale-Social Phobia Score	Mean (*SD*)	3.00(2.61))	2.62(2.44)	*t* (3252) = 3.46, *p* = .0006
Phobia Scale-Agoraphobia Score	Mean (*SD*)	2.47 (2.78)	2.18 (2.59)	*t* (3245) = 2.53, *p* = .012
Phobia Scale-Specific Phobia Score	Mean (*SD*)	2.19 (2.77)	1.92 (2.56)	*t* (3243) = 2.37, *p* = .018
**Post-treatment**				
Drop-out	Yes	270 (33.54)	642 (33.18)	0.98 (0.83 – 1.17), *p* = .85
	No	535 (66.46)	1293 (66.82)	1.0
Recovery	Yes	251 (33.07)	736 (42.08)	1.47 (1.23 – 1.76), *p* < .0001
	No	508 (66.93)	1013 (57.92)	1.0
RCSC	Yes	306 (42.03)	897 (49.49)	1.36 (1.14 – 1.61), *p* = .0006
	No	422 (57.97)	912 (50.41)	1.0

Numbers do not add up to totals due to missing data. Odds ratios (OR) represent the odds of being in the audit for each category of the presented variable relative to the reference category, denoted with OR = 1.0, e.g. the odds of being in the audit for those with anxiety relative to those with depression.

### Participants in the audit

Over a quarter of participants were classified as higher risk drinkers (28.30%) by scoring six or more on the AUDIT-C pre-treatment, including 36.17% of males and 24.39% of females. 15.32% scored eight or more on the AUDIT-C pre-treatment, including 21.28% of males and 12.33% of females.

For patients that had more than just an initial assessment session, there were no differences in the mean number of sessions attended for higher risk [mean (*SD*) = 4.79 (4.97)] and non-higher risk drinkers [mean (*SD*) = 4.67 (4.73)], *t* (3641) =  – 0.79, *p* = .43; although those above the higher cut-off of eight on the AUDIT-C [mean (*SD*) = 4.32 (4.93)] attended fewer sessions than those scoring below eight (4.85), *t* (3641) = 2.08, *p* = .038. Of those patients that had at least two treatment sessions, 642 (33.18%) dropped out before completing treatment.

### Primary and secondary outcomes

Logistic regression models were built to consider the associations between the two baseline AUDIT-C cut-offs and (i) drop-out from therapy, (ii) recovery, and (iii) reliable and clinically significant change on either the PHQ-9 or GAD-7 pre- to post-treatment after controlling for potential confounding factors (see Table 2).

**Table 2.** Number and percentage of participants experiencing each of the primary and secondary outcomes and the odds of the outcome by AUDIT-C cut-off pre-treatments

			OR	AOR			OR	AOR	RCSC		OR	AOR
AUDIT-C	Drop-out		(95% CI)	(95% CI)	Recovery		(95% CI)	(95% CI)	Change		(95% CI)	(95% CI)
score	*n* (%)		*p*-value	*p*-value*	*n* (%)		*p*-value	*p*-value†	*n* (%)		*p*-value	*p*-value‡
	**Yes**	**No**			**Yes**	**No**			**Yes**	**No**		
**<6**	447 (32.27)	938 (67.73)	1.0	1.0	510 (40.73)	742 (59.27)	1.0	1.0	627 (47.46)	694 (52.54)	1.0	1.0
**≥6**	195 (35.45)	355 (64.55)	1.15 (0.94 – 1.42), *p* = .18	1.19 (0.94 – 1.50), *p* = .15	226 (45.47)	271 (54.53)	1.21 (0.98 – 1.50), *p* = .07	1.22 (0.94 – 1.59), *p* = .14	270 (55.33)	218 (44.67)	1.37 (1.11 – 1.69), *p* = .0030	1.20 (0.89 – 1.63), *p* = .23
**<8**	526 (31.71)	1133 (68.29)	1.0	1.0	627 (41.97)	867 (58.03)	1.0	1.0	770 (49.33)	127 (51.21)	1.0	1.0
**≥8**	116 (42.03)	160 (57.97)	1.56 (1.20 – 2.03), *p* = .0007	1.60 (1.20 – 2.14), *p* = .001	109 (42.75)	146 (57.25)	1.03 (.79 – 1.35), *p* = .82	1.37 (0.98 – 1.92), *p* = .065	791 (50.67)	121 (48.79)	1.08 (0.82 – 1.41), *p* = .58	1.09 (0.75 – 1.59), *p* = .65

*Adjusted for: age, first PHQ-9 score, first GAD-7 score, employment status, first treatment step/intensity, prescribed psychotropic medications. ^†^Adjusted for: first PHQ-9 score, first GAD-7 score, first WSAS score, number of treatment appointments, and drop-out. ^‡^Adjusted for: first PHQ-9 score, first GAD-7 score, first WSAS score, prescribed psychotropic medications, number of treatment appointments, drop-out, and whether in recovery focused treatment or not.

There were no differences in the odds of the three outcomes for those scoring six or more compared with those scoring less than six on the AUDIT-C at baseline. However, those scoring eight or above on the AUDIT-C were at greater odds of dropping out of treatment [OR (95% CI) = 1.60 (1.20 – 2.14), *p* = .001], although scoring above this cut-off was not associated with recovery (*p* = .065) or RCSC (*p* = .65). There were no significant interactions between either cut-off and recovery or RCSC.

## Discussion

### Key findings

There were high rates of alcohol misuse in this sample with more than a third of male and a quarter of female patients higher risk drinkers. A little over one in five males and one in eight females met the higher cut-off for identifying higher risk drinkers. This compares with a 1-year prevalence of 8.9% in a UK general practice population (Aertgeerts et al., 2001). Higher risk drinking was not associated with the odds of dropping out from therapy, recovering post-treatment, or of experiencing reliable and clinically significant degrees of symptomatic improvement. Those scoring eight or more on the AUDIT-C were more likely to drop out of therapy but were no less likely to recover or experience reliable and clinically significant improvements pre–post treatment.

### Interpretation

The findings of this audit suggest that despite the high rates of alcohol misuse, IAPT services can adequately treat depression and anxiety disorders in people with comorbid alcohol problems as higher risk drinkers had no worse treatment outcomes. This is somewhat surprising as people with higher risk or dependent levels of drinking comorbid to common mental disorders have been found to have lower rates of remission from depression or anxiety disorders relative to people who have never had an AUD (Booschloo et al., 2012), and the lower threshold of ‘hazardous alcohol use’ has been found to be associated with poorer outcomes in one of the only studies of alcohol use and treatment outcomes in IAPT services (Giebel et al., 2014). However, the findings of the present study are in keeping with sections of the literature as previous studies have found that only severe alcohol dependence predicted worse treatment outcomes for patients with common mental disorders (Booschloo et al., 2012), and hazardous alcohol use was not associated with worse outcomes from internet-based CBT for patients with depression or social phobia (Gajecki et al., 2014).

The notion that drop-out might mediate the relationship between alcohol use and poorer treatment outcomes was generally not supported by this audit; however, patients scoring eight or above on the AUDIT-C were more likely to drop out. Quite why alcohol use was not associated with treatment outcomes is unclear. Findings from project MATCH and research into the ‘Hawthorne effect’ in alcohol use studies have shown that the majority of reduction in alcohol use occurs between assessment and starting treatment (PMR Group, 1998; McCambridge and Day, 2007), so perhaps the higher risk drinkers considerably reduced alcohol use once they had their IAPT assessment either because of the reactivity of the assessment or because they might have known they would soon be starting treatment for their depression/anxiety, negating the potential effect of their alcohol use on treatment outcomes because they experienced a lessening of depressive or anxiety symptoms pre-treatment associated to their reduced alcohol consumption. Selection bias based on alcohol use cannot be ruled out, particularly as only 69% of patients attending the service during the audit period completed the AUDIT-C questionnaire, and there were a number of small proportional differences between those that did and did not participate. It is plausible that patients referred to the IAPT services with the most severe levels of alcohol misuse may have either been identified based on referral information and referred on to specialist alcohol disorders services after a brief screening session but before having a full assessment in the IAPT service. In addition, patients may have been more likely to not complete the AUDIT-C if they did not want to make their clinicians aware of their alcohol problems and if this were to happen they would have been excluded from the present audit, or they may have disproportionately under-reported their levels of alcohol use relative to others who completed the audit. The three hypotheses above may be supported by the literature on access to mental health services whereby several studies have reported that patients with alcohol or other substance abuse disorders comorbid to common mental disorders are more likely to be turned down for treatment in specialist addiction and specialist mental health services, and patients with these comorbidities are more likely to have experienced negative staff attitudes towards them, influencing their decisions to access treatment (Fonseca et al., 2012; Tocque et al., 2011). The appropriateness of treating common mental disorders comorbid for AUD in IAPT may depend not on the severity of the alcohol misuse (if we accept that those with established dependence are unlikely to be referred to IAPT), but on the confidence and competence of the staff to work with such problems and on the timeline and relationship between the common mental disorder and the alcohol misuse.

### Limitations

There were several limitations to the present study. Being an audit, the sample was not randomly selected, so although regression models were built accounting for various potential confounding factors it is possible that unknown or unmeasured confounders may have impacted upon the results. Selection biases cannot be ruled out as it is plausible that a differential bias based on levels of alcohol use could exist and could have gone undetected as discussed above. Measurement error and other biases related to the use of a brief self-report instrument to measure alcohol use may have impacted some scoring, as self-report measures have frequently been found to under-estimate true levels of alcohol use (Boniface and Shelton, 2013). However, the sensitivity and specificity of the measure in identifying higher risk drinkers are very good (Aertgeerts et al., 2001) and it would not be appropriate or feasible to use more invasive methods (e.g. urine or blood based tests) in IAPT services. Further bias may have been introduced due to the nature of completing an assessment on alcohol use and the potential ‘Hawthorne effect’ brought about by doing so (McCambridge and Day, 2007). As the data for this audit come from just one IAPT service the findings may not generalize to other IAPT services in different locations with considerably different contextual and compositional demographic make-ups. Finally, the generalizability of these findings may be somewhat limited as the cut-offs on the AUDIT-C were not the same as the recent NHS England Commissioning for Quality and Innovation (CQUIN) framework advice (NHS England, 2017). The CQUIN has proposed that clinicians should identify and offer brief advice to patients defined as at ‘increasing risk’ of ill health due to drinking (based on scores of 5–7 on the AUDIT-C) and those at ‘higher risk’ (scores of 8–10 on AUDIT-C). The CQUIN guidance and associated target may have an impact on the way services attempt to identify and support service users that misuse alcohol, such that anyone scoring between 5 and 10 on the AUDIT-C might be offered advice on how to cut down their drinking and nothing more *per se*. However, as the impact of alcohol misuse on drop-out and treatment outcomes in an IAPT population had not been investigated before and the CQUIN advice is not based on validated cut-offs we chose to retain the two cut-offs (≥6 and ≥8) based on the psychometric properties of the AUDIT-C and the use of the measure to identify higher risk drinkers in the population sampled for this study.

### Clinical implications

This is one of the first investigations of alcohol use and its impact on treatment outcomes and drop-out in an IAPT population. Despite the limitations discussed above, the findings of this audit suggest that there are significant numbers of people with AUD comorbid to depression or anxiety attending IAPT services and they can access and benefit from IAPT treatment, although those scoring eight or above on the AUDIT-C may have greater difficulty accessing a full course of IAPT treatment.

Local policies might be adjusted to ensure people are not discriminated against from accessing IAPT services based on their alcohol use. Changes to local policy might include services aiming to routinely use the AUDIT-C with all patients during their first appointment, and for those scoring above five to then be given the full AUDIT questionnaire (Babor et al., 2001) which more thoroughly assesses alcohol dependence and the physical and mental consequences of risky drinking behaviours (e.g. blackouts and injuries). Additionally, clinicians might ask and consider the seven questions below which were developed to help clinicians move away from making ‘black and white’ decisions about whether a patient should be offered treatment within the IAPT service or referred on to a specialist alcohol disorders service based on presence or absence of alcohol misuse. Instead, they assist clinicians in evaluating the pattern and quantity of alcohol use and the impact of this on their ability to engage in treatment for depression or anxiety. The first five questions might be particularly relevant for patients between 5 and 10 on the AUDIT-C, and the last two will be particularly relevant for those scoring above 10:

(1) Is the main difficulty related to depressed mood, anxiety or PTSD?
(2) Is short-term IAPT therapy likely to be beneficial and manageable?
(3) Has the patient recently reduced their alcohol use?
  (a) If yes, have they reduced it incrementally over time?
    (i) If no, have they been physical well despite their reduction in use?
(4) If reduction in alcohol use has been slow, has the amount of use been stable for a period of time?
(5) Will being seen in IAPT contribute to their recovery away from alcohol misuse?

If currently or recently alcohol dependent:

(6) Can the patient attend sessions and complete homework without being intoxicated?
(7) Is the patient sober for most of the day?

If the answer to any of these questions is no, then clinicians should consider referral to specialist substance misuse services. If a patient scores highly on the AUDIT-C pre-treatment but the answer to all relevant questions is yes, then we suggest the use of identification and brief advice (IBA), which is an intervention for alcohol use based on motivational interviewing principles (NICE, 2011), and then treatment as usual for the common mental disorder within the IAPT services. In addition, to improve engagement and manage expectations we suggest that clinicians ask all patients to complete a treatment contract which (when appropriate) would include an agreement that they will attend sessions sober, and that the clinician will cancel the session if they attend intoxicated. Clinicians should advise patients on how to reduce their alcohol intake by cutting down the amount and (where applicable) the strength of the alcohol consumed, and when possible encourage patients to abstain from using alcohol, particularly whilst engaging in behavioural interventions. Joint working and regular communication with specialist alcohol disorder services is to be encouraged and their input sought when considering harm reduction for alcohol-dependent patients; if patients are receiving support from such a service, this might be seen as an advantage rather than as a barrier to accessing psychological treatment in IAPT. A range of interventions can be offered by IAPT services for patients with AUD including identification and brief advice, pure or guided self-help for alcohol misuse, incorporating alcohol use into high-intensity treatments for depression or anxiety (e.g. combining CBT with some principles of motivational interviewing), or offering interventions focused on linking patients with services that offer support for social, financial and other difficulties contributing to or affected by the patient's alcohol misuse. Joint-working protocols could also invite alcohol disorder services to re-refer to IAPT for subsequent therapy for depression or anxiety following detox, and potentially continue to offer input or advice around alcohol misuse relapse prevention.

### Conclusions

IAPT services are established nationwide in England, and a number of other countries are now setting up psychological treatment services based on the IAPT model. This audit suggests that the population of patients attending these services might include significant numbers with higher risk drinking behaviours, particularly among male patients. Alcohol misuse did not impact upon treatment outcomes so IAPT services might be well placed to offer evidence-based psychological interventions for depression and anxiety to people misusing alcohol. AUDIT-C scores should not be a marker for excluding patients from IAPT. Patients scoring eight or above on the AUDIT-C pre-treatment were more likely to drop out from therapy so it is important that guidance is given that might help improve engagement in therapy and set clear expectations and boundaries on the therapeutic relationship that are particularly relevant when offering treatment to patients in this context. In order for IAPT interventions to be conducted effectively with patients that misuse alcohol, it is important that IAPT staff are appropriately trained in the assessment and brief treatment of alcohol misuse, and are well supported to do this in supervision.
